# Novel Growth Hormone-Releasing Hormone Receptor Gene Mutations in Turkish Children with Isolated Growth Hormone Deficiency

**DOI:** 10.4274/jcrpe.1518

**Published:** 2014-12-05

**Authors:** Ahmet Arman, Bumin Nuri Dündar, Ergun Çetinkaya, Nilüfer Erzaim, Atilla Büyükgebiz

**Affiliations:** 1 Marmara University Faculty of Medicine, Department of Medical Genetics, İstanbul, Turkey; 2 Katip Çelebi University Faculty of Medicine, Department of Pediatric Endocrinology, İzmir, Turkey; 3 Endomer Pediatric Endocrinology Center, Ankara, Turkey; 4 Yeditepe University Faculty of Medicine, Department of Genetics and Bioengineering, İstanbul, Turkey; 5 Bilim University Faculty of Medicine, Department of Pediatric Endocrinology, İstanbul, Turkey

**Keywords:** IGHD, GHRHR gene, short stature

## Abstract

**Objective:** Isolated growth hormone deficiency (IGHD) is defined as a medical condition associated with growth failure due to insufficient production of GH or lack of GH action. Mutations in the gene encoding for GH-releasing hormone receptor (GHRHR) have been detected in patients with IGHD type IB. However, genetic defects on GHRHR causing IGHD in the Turkish population have not yet been reported. To identify mutations on GHRHR gene in a population of Turkish children with IGHD.

**Methods:** Ninety-six Turkish children with IGHD were included in this study. Exon1-13 and exon/intron boundaries of GHRHR were amplified by suitable primers. The polymerase chain reaction products for GHRHR gene were sequenced with primers.

**Results:** We analyzed the GHRHR gene for mutations in ninety-six patients with IGHD based on sequence results. We identified novel p.K264E, p.S317T, p.S330L, p.G369V, p.T257A and C base insertion on position 380 (c.380inserC) mutations. In 5 of the patients, the mutation was homozygote and in 1-heterozygote (p.S317T).

**Conclusion:** Six new missense mutations and one first case of insertion mutations for the GHRHR gene are reported.

## INTRODUCTION

Growth hormone (GH) is a 22 kDa protein involved mainly in skeletal and visceral growth but also in carbohydrate, protein and lipid metabolisms (1). GH is synthesized and secreted by somatotropes in the anterior pituitary gland. The expression and secretion of GH are regulated multifactorially, but predominantly by hypothalamic hormones, GH-releasing hormone (GHRH), GH secretagogue (GHS) and somatostatin (SS) (2).

GH deficiency (GHD) is defined as deficient or insufficient production/secretion of GH from the pituitary gland (3,4,5). The prevalence of short stature associated with GHD is between 1/4000 and 1/10 000 live births (6,7). Although most of the cases are sporadic and thought to be caused by environmental cerebral insults or developmental anomalies, 5-30% of cases are familial (8).

Based on their severity and mode of inheritance, there are three types of familial isolated GHD (IGHD) (9). While types 1 A and 1 B show a recessive autosomal transmission, type 2 shows an autosomal dominant transmission. Type 3 shows an X-linked chromosome pattern. Patients with type 1 A have severe short stature, they lack any detectable GH and generally produce GH antibodies. These cases are mainly caused by deletion of the entire GH (GH-1) gene (10). Patients with type 1 B are milder and they respond to GH treatment very well. IGHD type 1 B is caused by mutations in both GH-N gene which is one of the GH-gene clusters (hGH-N, hCS-L, hCS-A, hCS-B and hGH-V) encoding 22 kDa GH protein and GHRH receptor (GHRHR) gene (2,11,12,13).

GHRH, through GHRHR, plays an important role in GH expression and secretion (14,15). The human GHRHR gene is located on the short arm of chromosome 7, is mostly expressed in the anterior pituitary gland and belongs to a G protein-coupled receptor superfamily (16). The GHRHR gene consists of 13 exons and encodes a 423-amino acid protein with an N-terminal and a C-terminal domain linked by 7 alpha-helical transmembrane domains (16).

More than 20 mutations for GHRHR have been reported in patients with IGHD; homozygous and compound heterozygous mutations lead to a loss of GHRHR function. These are missense, splice, nonsense, microdeletion and promoter mutations (17,18,19,20,21).

In this study, we analyzed the GHRHR protein coding region and the exon/intron boundary of the GHRHR gene for mutations in 96 children with IGHD.

## METHODS

A total of 96 patients with IGHD (59 boys, 37 girls) were included in this study; 6 of these patients had GHRHR mutations. At least two GH stimulation tests were performed in each patient. Height and weight standard deviation scores (SDS) were calculated according to standard reference values for age, sex and pubertal maturation. GH stimulation tests (ITT and L-DOPA) were performed (deficiency defined as a GH peak <10 ng/mL) and other pituitary hormone deficiencies were ruled out by measuring free thyroxine (fT4) and cortisol levels. Serum GH levels were measured by RIA or ELISA, insulin-like growth factor-1, insulin-like growth factor binding protein-3 levels were determined by immunoradiometric assays (22). FT4 and thyroid-stimulating hormone levels were assessed on the AxSYM system by microparticle enzyme immunoassay and cortisol was measured using a chemiluminescence immunoassay. Clinical and hormonal data of the 6 children identified to have GHRHR gene mutations are shown Table 1.

**DNA Isolation and Specific Exon and Exon/Intron Boundary Polymerase Chain Reaction (PCR)**

**Figure 1:**
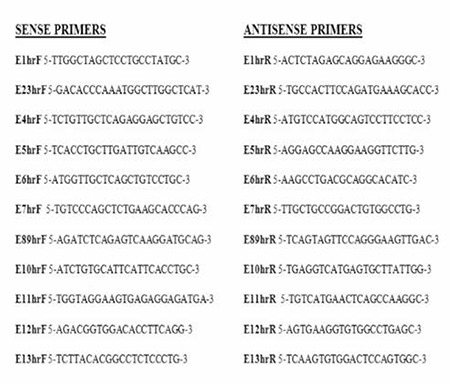
Primers for PCR. The primers for GHRHR were used for the amplifications of specific exons and exon/intron boundaries for exons 1-13. F shows forward primer and R refers reverse primer

Genomic DNA was isolated from bloods of children with IGHD based on salting out method (23). Exon 2-3 and 8-9 together and other exons (1,4,5,6,7,**10**,^11^,^12^,^13^) alone and their flanking splice sites were amplified by PCR using the exon-specific primers shown in Figure 1. PCR products were visualized on agarose gels to rule out large deletion and insertions.

**DNA Sequencing**

The PCR products of GHRHR exons for each patient were purified and sequenced with direct sequencing of the DNA Cycle Sequencing System (ABI Prism kit) with the dideoxychain termination method and applied on an autosequencer (ABI Prism 377 DNA sequencer).

**Silico Functional Analysis**

PolyPhen-2 and PROVEAN programs were used in order to predict the function of mutated amino acids on the GHRHR protein structure.

**Ethics Approval**

This research was approved by the ethics committee of Marmara University. All human rights were protected and any necessary approval was secured from the ethics committee. Written informed consent was taken from the parents of each patient.

## RESULTS

Ninety-six children were diagnosed as IGHD by at least two GH stimulation tests; they presently are under GH treatment. Six of the patients had GHRHR mutations and prevalence of mutation in this series was 6.25%. A summary of mutations on the GHRHR gene in these 6 children with IGHD is shown in Table 2.

**Figure 2:**
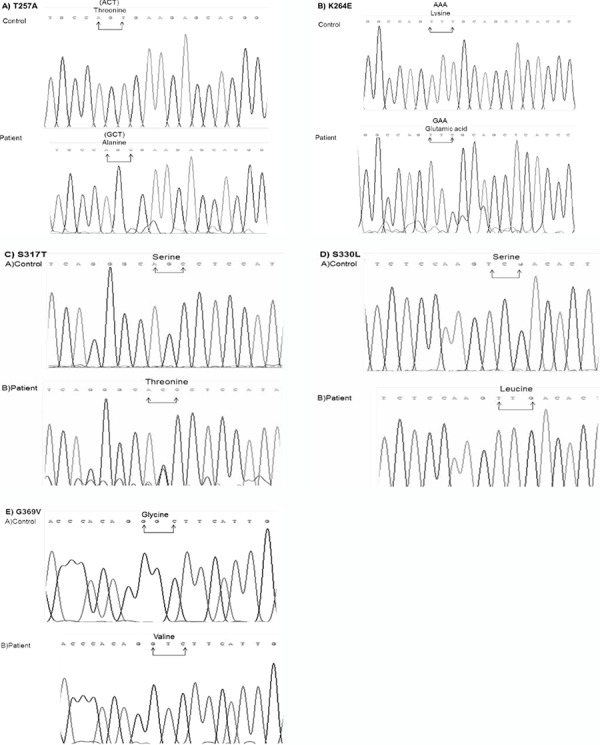
Determination of missense GHRHR gene mutations. Sequence analysis of A) T257A Mutation, Normal person carries Threonine (T) amino acid residues on position 257 in GHRHR gene encoded by ACT. The A residue of ACT was changed to G (GCT) encoding Alanine (A) in IGHD patient (sequence from reverse), B) K264E Mutation, Lysine (K) is encoded by AAA in the healthy person and the first A residue of AAA codon was converted to G (GAA) in the patient (reverse sequences), C) S317T Mutation, healthy person has AGC codon encoding Serine (S) and Threonine (T) occurred by changing of G residue of AGC codon to C (ACC) in IGHD patient, D) S330L Mutation, The healthy person has Serine (S) amino acid on position 330 encoded by TCG and Leucine (L) mutation was created by substitution of the C residue of TCG to T (TTG) in the patient, E) G369V Mutation, the control has GGC codon encoding Glycine (G), but this amino acid is changed to Valine (V) by the substitution of the second G residue of GGC to T (GTC) in the patient

Five different missense mutations were determined in the exons 8, 10, 11 and 12 of the GHRHR gene. The p.T257A homozygous mutation was located at exon 8 and was seen in patient 9. This mutation was created by the substitution of the A residue of ACT codon encoding threonine (T) to G and GCT codon encodes alanine (A) (Figure 2A). p.K264E mutation seen in patient 50 was homozygous, located at exon 8 and was created by replacement of the first A residue of AAA encoding lysine (K) to G and GAA codon encoding glutamic acid (E). This mutation is novel (Figure 2B). Another missense mutation was found in patient 51 - p.S317T located in exon 10. This mutation occurred by changing of G residue of AGC codon to C (ACC) (Figure 2C) and is a heterozygous mutation. p.S330L mutation is located at exon 11 and was seen in patient 21. This mutation was created by changing the C residue of TCG to T residue (TTG) (Figure 2D). The last missense mutation was p.G369V mutation located at exon 12 and observed in patient 40. This mutation was created by substitution of the second G residue of GGC to T (GTC) (Figure 2E).

**Figure 3:**
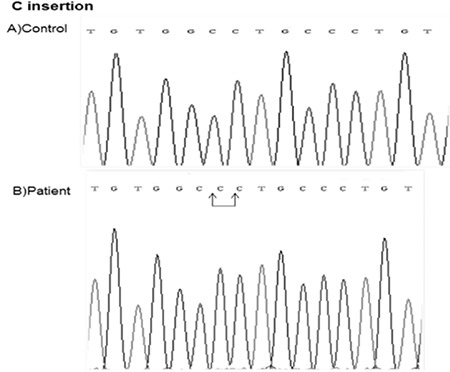
Determination of C insertion mutation at exon 4 of GHRHR gene. A shows the sequence from normal person, B shows C insertion at 380 residue located at exon 4 of GHRHR in the patient

Another interesting and rare mutation found in this Turkish population was C insertion at position 380 residue (c.380inserC) - a homozygote mutation located in exon 4 which was found in patient 52 (Figure 3). This mutation changed Open Reading Frame of GHRHR and introduced stop codon (p.C112Lfs*9).

Furthermore, we found two sense mutations p.Y163Y and p.L247L (Table 2). Also, we found two polymorphisms, p.A57T and p.E121D, located in exons 3 and 4, respectively in Turkish population (Table 2). The homozygote p.A57T polymorphism was found only in patient 40 containing p.G369V mutation (Table 2); however, the homozygote pE121D polymorphism was seen in patient 21 containing p.S330L mutation and in patients 32 and 50 containing p.K264E mutation,78 (Table 2). p.A57T and p.E121D polymorphisms were reported previously. However, p.K264E, p.S317T, p.S330L, p.G369V, p.T257A and C insertion at exon 4 mutations are novel.

## DISCUSSION

GHRH induces GH expression and secretion through GHRHR (24). Although GHS, also known as ghrelin, is involved in GH secretion via GHS receptor (GHSR), somatostatin suppresses GH secretion in the pituitary. Deficiency of GH secretion leads to growth failure and to changes in metabolic activities. Mutations in GH-1 cause mainly types 1 and 2 of IGHD; however, they have been determined only in less than 10% of total cases and in less than 2% of patients with type 1 B IGHD suggesting the involvement of other genes. GHRH is supposed to be involved in GH secretion; however, no mutation was detected in GHRH gene. It has been reported that mutations in GHRHR gene are being detected with increasing frequency. More than 20 GHRHR mutations were reported to date, including missense mutations (13,25,26,27,28,29), nonsense mutations (13,25,29,30,31), splice site mutations (12,29,32,33,34,35,36,37,38,39), promoter mutation (20), micro-deletion mutations (19,26,40) that lead to loss of GHRHR function. The missense mutations show defective ligand binding resulting in blocking GHRH-GHRHR signaling (33); promoter mutations affect promoter activity and thus change GHRHR expression (26); nonsense and splicing mutations produce truncated GHRHR receptor and can be involved in mRNA instability (29).

We identified five different missense (p.K264E, p.S317T, p.S330L, p.G369V and p.T257A) and one insertion mutation (C insertion) on the GHRHR gene from 96 IGHD patients. We also found p.A57T and p.E121D polymorphisms in GHRHR, reported previously. p.K264E, p.S317T, p.S330L, p.G369V, p.T257A and C insertion at exon 4 mutations are novel.

One of missense mutations in GHRHR is p.T257A homozygous mutation seen in patient 9 (Figure 2A, Table 2). This mutation occurred on the fourth transmembrane domain of GHRHR and probably causes neutral effect on the receptor based on functional silico analysis. We do not have functional analysis of this mutation for GHRHR signaling in the cell culture system, but this mutation is novel. It has been reported that CHO cells expressing p.F242C mutation on the same transmembrane domain of GHRHR did not alter the receptor surface expression, but the authors failed to show cAMP production after treatment of cells with GHRH (41). This result showed that mutation on the transmembrane domain affects GHRHR-GHRH binding affinity.

We observed homozygous p.K264E missense mutation, a finding not reported previously; thus, it is a novel mutation. This mutation occurred at the extracellular loop of the transmembrane domain 4 of GHRHR; it is probably detrimental for receptor based on silico functional analysis and may reduce the ligand binding to receptor, impairing the receptor ability to transmit intracellular signaling.

We also determined heterozygous p.S317T missense mutation that lied within the intracellular loop of the sixth transmembrane domain of GHRHR. This mutation has a damaging effect according to the PolyPhen-2 program. It is interesting that this is a heterozygote mutation, since most GHRHR mutations are inherited in an autosomal recessive way. Heterozygote mutations on GHRHR have been reported previously (12,18,26). The mother of the patient carrying S317T mutation was of short stature but not the father. However, we do not have any data on the genetic make-up of this patient’s parents. This mutation can change the structure of GHRHR and may lead to defectiveness of intracellular signaling. There is no reported mutation in this region of GHRHR receptor so far.

Another homozygous missense mutation is p.S330L mutation located on the sixth transmembrane domain of GHRHR. This mutation will have a deleterious effect on the receptor since polar amino acid (S) was converted to non-polar amino acid (L). This mutation probably has a reducing effect on GHRH binding to GHRHR, leading to a block in intracellular signaling. Another mutation called p.K329E and encoding an amino acid for GHRHR was also reported. Chinese hamster ovary cells transfected with cDNA encoding p.K329E GHRHR failed to induce cAMP production (20). This experiment showed that this mutation completely knocked out the function of GHRHR.

The last missense mutation is a p.G369V mutation located at the seventh transmembrane domain of GHRHR. This mutation is detrimental based on functional silico analysis. This mutation probably destroys GHRHR structure resulting in the inhibition of GHRH binding to GHRHR and thus disrupts GH signaling. A c.1120-1123delATCC mutation was reported in this transmembrane domain GHRHR (40) and four base pair deletion in exon 12 of the GHRH receptor caused a frameshift and the premature stop codon resulted in a C-terminally truncated GHRHR receptor that blocked GHRH-GHRHR signaling.

GH levels after GH-stimulation of patients 9 and 50 were 8.3 and 8.07 ng/mL, respectively. These levels are close to normal post-stimulation GH levels (10 ng/mL). Both mutations (p.T257A and p.K264E) are located at the GHRHR gene, but the biological activity of the receptor is different because of a different effect of the mutation on GHRHR or because of a possibly existing different domain of receptor. Height SDS of both patients (patients 9 and 50) was -4.6 and -1.77, respectively. The lower height SDS of patient 50 may be due to an additional effect of the polymorphism (p.V121D) noted in patient 50.

In patient 52, we detected a homozygote C insertion (c.380inserC) on position 380 which was located at the extracellular domain of GHRHR (Figure 3, Table 2). This insertion causes frameshift mutation (p.C112Lfs*9) that introduces stop codon (after changed 9 amino acid coding sequence) proceeding sequence of GHRHR gene. The mutated receptor is missing of the part of exon 4 and exons 5-13 and this mutation is also novel. It is interesting that there are no reports on insertion mutation on GHRHR gene to date. The function of novel mutations is under investigation.

Additionally, we identified two heterozygote sense mutations in this Turkish population which are p.Y163Y located at exon 6 and p.L247L found at exon 7 (Table 2). We do not know whether or not these mutations affect codon usage in the human.

Furthermore, we detected homozygote p.A57T and p.E121D polymorphisms reported previously (42,43). It has been reported that TSA cell lines carrying p.A57T and p.E121D mutations did not induce cAMP production in absence of GHRH but induced cAMP production in presence of GHRH (43). This result showed that these polymorphisms may not affect GHRHR function or the pathogenesis of IGHD. However, it has been reported that the tumors containing p.A57T polymorphism responded strongly to GHRH-based cAMP production in vitro (42).

In conclusion, we report five missense and one insertion mutations determined in a Turkish population consisting of 96 children with IGHD. These mutations are novel. These mutations are probably unique for Turkey since previously reported mutations were located in unique geographic locations.

**Acknowledgement**

This project was supported by a grant from the Scientific and Technical Research Council of the Turkish Republic.

## Figures and Tables

**Table 1:**
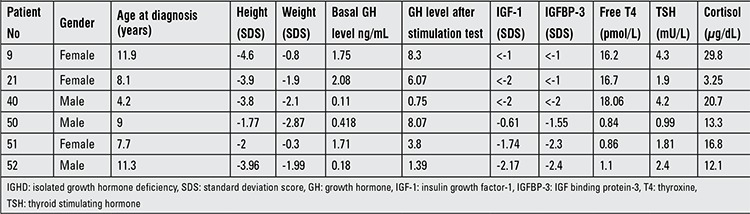
Clinical and hormonal data of six patients with IGHD

**Table 2:**
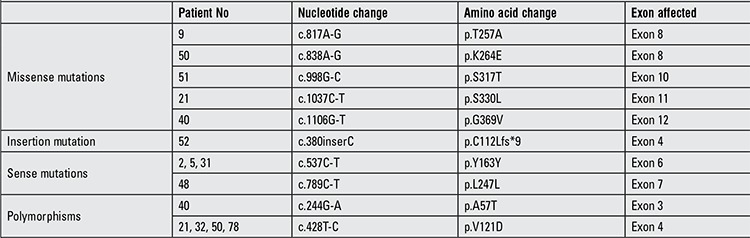
Growth hormone-releasing hormone receptor (GHRHR) mutations encountered in this series of Turkish children
